# The Unique Metabolic Characteristics of Bone Marrow Adipose Tissue

**DOI:** 10.3389/fendo.2019.00069

**Published:** 2019-02-08

**Authors:** Yujue Li, Yang Meng, Xijie Yu

**Affiliations:** ^1^Laboratory of Endocrinology and Metabolism, Department of Endocrinology and Metabolism and National Clinical Research Center for Geriatrics, West China Hospital, Sichuan University, Chengdu, China; ^2^Department of Orthopedics, West China Hospital, Sichuan University, Chengdu, China

**Keywords:** marrow adipose tissue, bone marrow, endocrine, bone mesenchymal stem cell, adipokine

## Abstract

Bone marrow adipose tissue (MAT) is distinct from white adipose tissue (WAT) or brown adipose tissue (BAT) for its location, feature and function. As a largely ignored adipose depot, it is situated in bone marrow space and resided with bone tissue side-by-side. MAT is considered not only as a regulator of bone metabolism through paracrine, but also as a functionally particular adipose tissue that may contribute to global metabolism. Adipokines, inflammatory factors and other molecules derived from bone marrow adipocytes may exert systematic effects. In this review, we summary the evidence from several aspects including development, distribution, histological features and phenotype to elaborate the basic characteristics of MAT. We discuss the association between bone metabolism and MAT, and highlight our current understanding of this special adipose tissue. We further demonstrate the probable relationship between MAT and energy metabolism, as well as glucose metabolism. On the basis of preliminary results from animal model and clinical studies, we propose that MAT has its unique secretory and metabolic function, although there is no in-depth study at present.

## Introduction

Adipose tissue, distributed in distinct depots in the whole body, may affect overall health through endocrine function. White adipose tissue (WAT), as an energy-storing reservoir, principally locates in the subcutaneous and visceral depots; while brown adipose tissue (BAT), specializing in utilizing energy to produce heat, is primarily present above the clavicle and in the subscapular region of the back (1, 2). In addition to brown adipocytes, brite/beige adipocytes, also expressing uncoupling protein (Ucp) 1, principally store lipids and can be stimulated to transdifferentiate into a “brown-like” state with well characterized-thermogenic function (2–4). The brite/beige fat has been discovered in WAT in response to activators like cold exposure, indicating the involvement of sympathetic signaling (5, 6). The adipocytes also exist in the bone marrow (BM), and such marrow adipose tissue (MAT) accounts for over 10% of total adipose tissue mass in humans (7, 8). MAT has long been considered as a relatively inert and underappreciated component in the BM microenvironment but it has been recognized recently to have potentially significant and diverse functions.

The tenet widely accepted is that the amount of MAT is increased with age, obesity, and some metabolic disorders (9, 10). As the adipose tissue is one of the main components within the BM niche, there is a need to characterize the properties and functions of MAT. Previous review literatures mainly focused on the relationship between MAT and bone metabolism (11)/hematopoiesis (12). Although it has been summarized the ability of MAT to secrete traditional adipokines (7, 13) (including leptin and adiponectin), this review, combining the latest reports, has discussed the regulation of other small molecules derived from MAT, including inflammatory factors and cytokines. In addition, we have summarized the current evidence regarding the fundamental features and regulatory factors of MAT and discussed the inner relationships between MAT and some metabolic disorders.

## The Basic Characteristics of MAT

### Development of MAT

BM, primarily consisting of adipocytes and hematopoietic red blood cells (red marrow), is located in the cavities of trabecular bone. At birth, BM cavities mainly contain active hematopoietic red marrow. Then, MAT accumulates centripetally in a time-dependent way: the process starts from the terminal phalanges, continues to the appendicular skeleton and finally appears in the axial skeleton (14, 15). Significantly, this depot makes up ~50–70% of the marrow volume by the age of 25 and accounting for over 10% of total adipose mass in lean, healthy adults (8, 16–18). Afterwards, the BM transforms slowly into MAT throughout the rest of lifetime.

### Distribution of MAT

Deep analysis of the BM requires researchers to deal with the anatomy of bone. This explains why most of the initial work on BM in the late 1800s and early 1900s was relied on the relatively larger animals like rabbits or cats (19–22), so as to appropriately observe and analyze anatomical structure of BM. The existence of MAT, traditionally considered to be yellow marrow, has been neglected for many decades although it was mentioned in the early literature as a long-standing knowledge (23). However, one such cell population known as MAT or yellow marrow has attracted increasing attention by the scientific community recent years (24–26). In 1976, Tavassoli began to characterize the marrow adipocytes and delineate their morphologic features (24). Two histochemically distinct populations of fat cells, one is presented within the red marrow staining positively with performic acid-Schiff (PFAS) and the other is located in the yellow marrow non-staining with PFAS (24). This staining reaction is considered to rely on the oxidation of ethylene to acetaldehyde in the unsaturated fat and treatment with Schiff's reagent to produce a red/purple color. PFAS-positive adipocytes in red marrow disappeared in response to experimentally induced hemolysis, while non-stained adipocytes remain unaffected (25). This implies that red marrow with PFAS-positive cells is mainly composed of unsaturated lipids, while yellow marrow with PFAS-negative cells primarily consists of saturated lipids.

**Figure 1:**
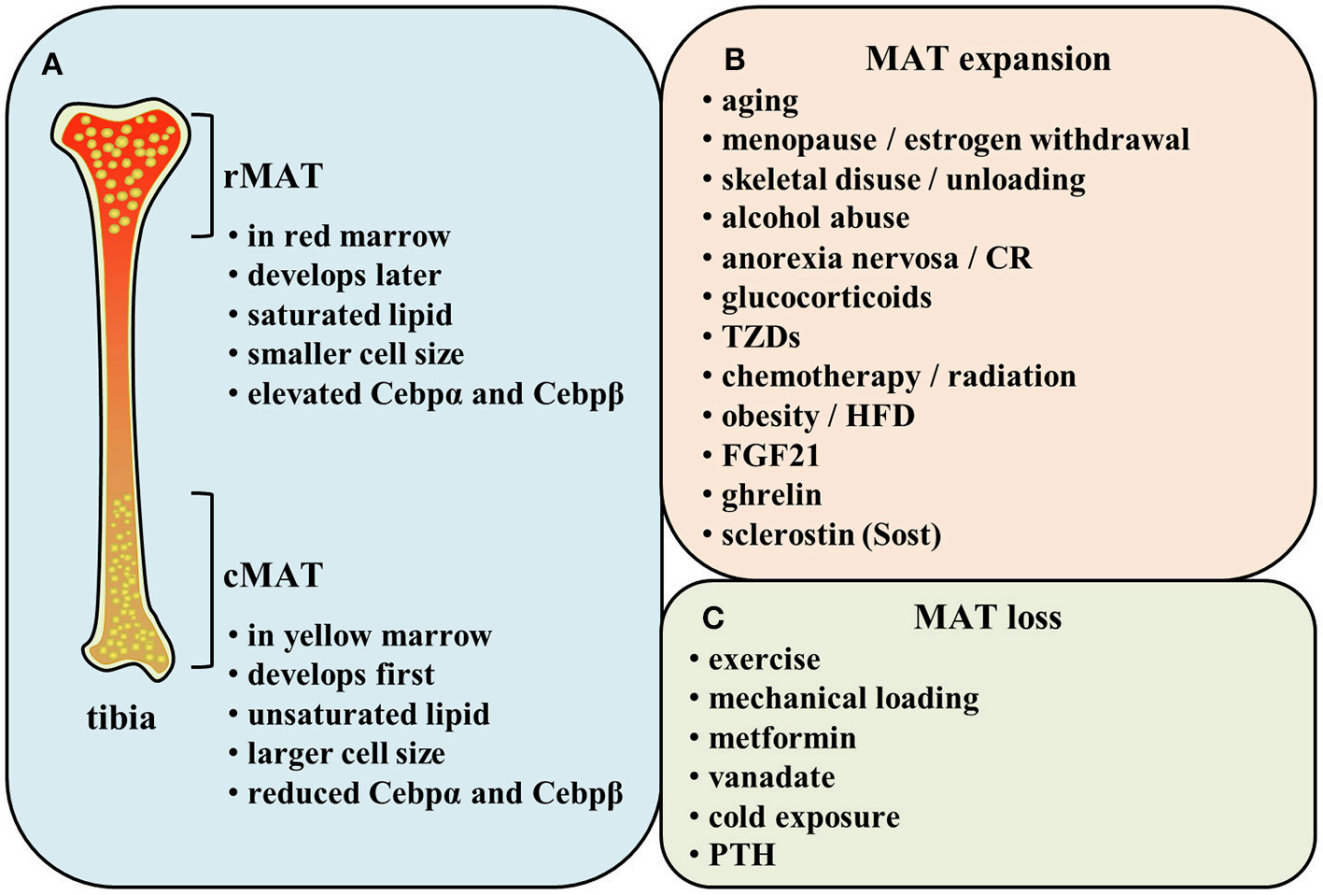
**(A)** Comparison of rMAT and cMAT. **(B,C)** Regulatory factors of MAT.

Indeed, 40 years later, Scheller and collaborators demonstrated a different distribution of lipid saturation in the BM by proton MRS (^1^H-MRS) from the findings of Tavassoli. MAT arisen first and early in life in the distal skeleton (e.g., distal tibia and caudal vertebrae) is identified as constitutive marrow adipose tissue (cMAT) within the yellow marrow. The other subtype of MAT exists in the lumbar/thoracic vertebrae, proximal limb skeleton, hip, and ribs, which is formed late and in a more scattered way. This type of MAT is known as regulated marrow adipose tissue (rMAT) within the red marrow. Researchers have found that distal marrow adipocytes (cMAT) contain more unsaturated lipids than those in proximal/central skeletal regions (rMAT) (26) (Figure 1A). This implies that the populations of rMAT fail to stain with PFAS while their constitutive counterparts readily display the characteristic pattern of PFAS staining (24, 27). Of note, this study yielded a conclusion of the increase in cMAT unsaturation which is opposite to the theory based on the proposed mechanism of PFAS staining. Nevertheless, there is possibility that both regulated and constitutive MAT adipocytes exist in the same position (26). It is possible that the rMAT can be matured into the stable cMAT in some conditions.

### Histological Features of MAT

Similarly to WAT, MAT pre-adipocytes accumulate lipid that merges into a unilocular droplet, replacing nucleus and cytoplasm gradually (14, 28). The average MAT adipocyte diameter is measured and calculated to be 30–40 mm through osmium-based MAT analysis (26). BAT and brite/beige adipocytes are smaller and occupied by high mitochondrial content, as well as multilocular lipid droplet (29), while neither of which is observed in BM adipocytes (8, 30). Analysis of C3H mice and Sprague-Dawley rats demonstrated that cMAT adipocytes are significantly larger than rMAT adipocytes (26). MAT in diet-induced obesity mice show greater adipocyte size and number, while both measures are reduced with exercise (31–33).

### Phenotype of MAT

It has been hypothesized that the metabolic profile of MAT resembles both BAT and WAT. On the one hand, MAT is assumed to have a similar feature to BAT, as the volume of MAT is adjusted by temperature (34). Compared with BAT, the entire tibia bones express higher BAT-specific gene markers, including modulators of thermogenesis such as deiodinase (Dio) 2 and peroxisome proliferator-activated receptor-gamma coactivator (Pgc) 1α, as well as transcription factor positive regulatory domain-containing (Prdm) 16 in C57BL6/J mice (35). However, the expression of Ucp1 in tibia was not suggested to be higher. Sulston et al. demonstrated that antidiabetic thiazolidinedione (TZD) rosiglitazone upregulated Ucp1 in BAT, but not in MAT of tibia (30), revealing that MAT of tibia may not share the thermogenic properties. Moreover, Ambrosi et al. indicated that the gene expression patterns of MAT in tibia are similar to inguinal WAT (iWAT) rather than BAT. MAT expresses similar levels of peroxisome proliferator-activated receptor (Ppar) γ and CCAAT/enhancer-binding protein (Cebp) α and low level of Ucp1 (36). However, high expression of Ucp1 was found in vertebral BM in humans and ICR mice (37). Taken together, the seemingly contradictory results tend to suggest that the potential presence of active brite/beige adipocytes in MAT of vertebrae in mammals. That means, marrow adipocytic lineage may possess the BAT-like thermogenic properties in vertebrae, but often exhibits a WAT-related phenotype in tibia.

Additionally, both in human and rodent bones, cMAT is reported to express elevated levels of the adipogenic transcription factors (e.g., Cebpα and Cebpβ) and be principally composed of unsaturated lipids. Conversely, rMAT is described to express reduced Cebpα and Cebpβ and be mainly constituted of saturated lipids (26, 28) (Figure 1A). These evidences imply that constitutive and regulated MATs not only differ in their position but also exhibit distinct features in their function. Since the time for identification of MAT classification is not long, there still remains much unclear about cMAT and rMAT. It is unknown whether they originate from a common progenitor or not and how the microenvironment affects their development. In addition, we also need to explore the effect of each subtype on metabolism not only in bone turnover but also in the whole body during some specific physiopathological conditions.

## Regulatory Factors of MAT

### MAT Expansion

MAT alters its volume in order to adapt to various physiologic and pathologic conditions. Indeed, MAT is a potentially critical participant in bone homeostasis since osteoblasts and adipocytes are derived from the same common progenitor cells-bone marrow mesenchymal stem cells (MSCs) (38–41). Marrow adiposity is correlated with low bone mass, indicating that the decision for MSCs to differentiate into osteoblasts or adipocytes may create a tug-of-war between MAT and bone tissue (42, 43). Many osteoporotic states in humans, including aging (36, 44–46), menopause (47, 48), skeletal disuse or unloading (49, 50), alcohol abuse (51), and anorexia nervosa (52, 53), are associated with increased bone marrow adiposity, suggesting the balance between MAT and bone mineral density (BMD) has been broken. Researchers have also shown that glucocorticoids (18), TZDs (30, 31, 41, 54–59), caloric restriction (8, 18, 60), chemotherapy and/or radiation (8), obesity (36, 45, 61), high-fat diet (HFD) (33, 61–64), and hormonal factors such as estrogen withdrawal (46, 48), fibroblast growth factor (FGF) 21 administration (65) are related to a significant increase in MAT. However, increased marrow fat led by HFD or obesity is coincided with preserved or increased bone density (33, 61–64). Thompson et al. also demonstrated that des-octanoyl ghrelin, a major circulating form of ghrelin, promotes BM adipogenesis *in vivo* by a direct peripheral action (66). Besides, a recent work demonstrates a direct role for sclerostin (Sost), secreted from osteocytes, to induce BM adipogenesis through inhibiting Wnt signaling (67) (Figure 1B). It has been reported that inhibition of Wnt signaling increased expression of adipogenic transcription factors Pparγ and Cebpα and stimulated adipogenesis (68–70). Levels of mRNA expression adipogenesis markers Pparγ2, lipoprotein lipase (LPL), adipocyte-specific fatty acid binding protein (aP2), and adiponectin were lower when incubated with adipocytes induction medium containing wnt3a than without wnt3a (71). Osteocyte-derived Sost induced adipogenesis in mouse primary bone marrow MSCs, increased the expression of Pparγ and Cebpα, and simultaneously decreased the expression of β-catenin responsive genes Axin2 and Smad6 (67). The above results demonstrate Wnt signaling inhibits adipogenic differentiation of mouse MSCs and human MSCs, and Sost derived from osteocytes could inhibit Wnt signaling, thus promoting adipogenesis in BM.

### MAT Loss

The exercise or mechanical loading have been reported to lower MAT volume (31, 33, 57, 63, 64, 72–75). The exercise can reduce MAT adipocytes in both lean and obese mice (33). Moreover, metformin, the most widely prescribed medicine for type 2 diabetes (T2D) worldwide, ameliorates elevated MAT induced by HFD in tibia (61). Besides, vanadate impedes adipogenesis significantly in MSCs within BM (76). A recent study revealed that proximal rMAT adipocytes are decreased in size and number in response to cold exposure (26). Some endocrine signals like parathyroid hormone (PTH) also strongly influence the extent of MAT. Fan et al. found MAT increased through conditional deletion of the PTH/PTHrP receptor (PTH1R) in MSCs using Prx1-Cre recombinase (77). Moreover, intermittent PTH administration can effectively reduce the increased marrow fat in mice and osteoporotic patients (77, 78) (Figure 1C). Therefore, many regulatory factors lead to the changes of MAT. This reflects the strong plasticity of MAT and implies its vital functions.

## Secretory Property of MAT

### Extracellular Vesicles

**Figure 2:**
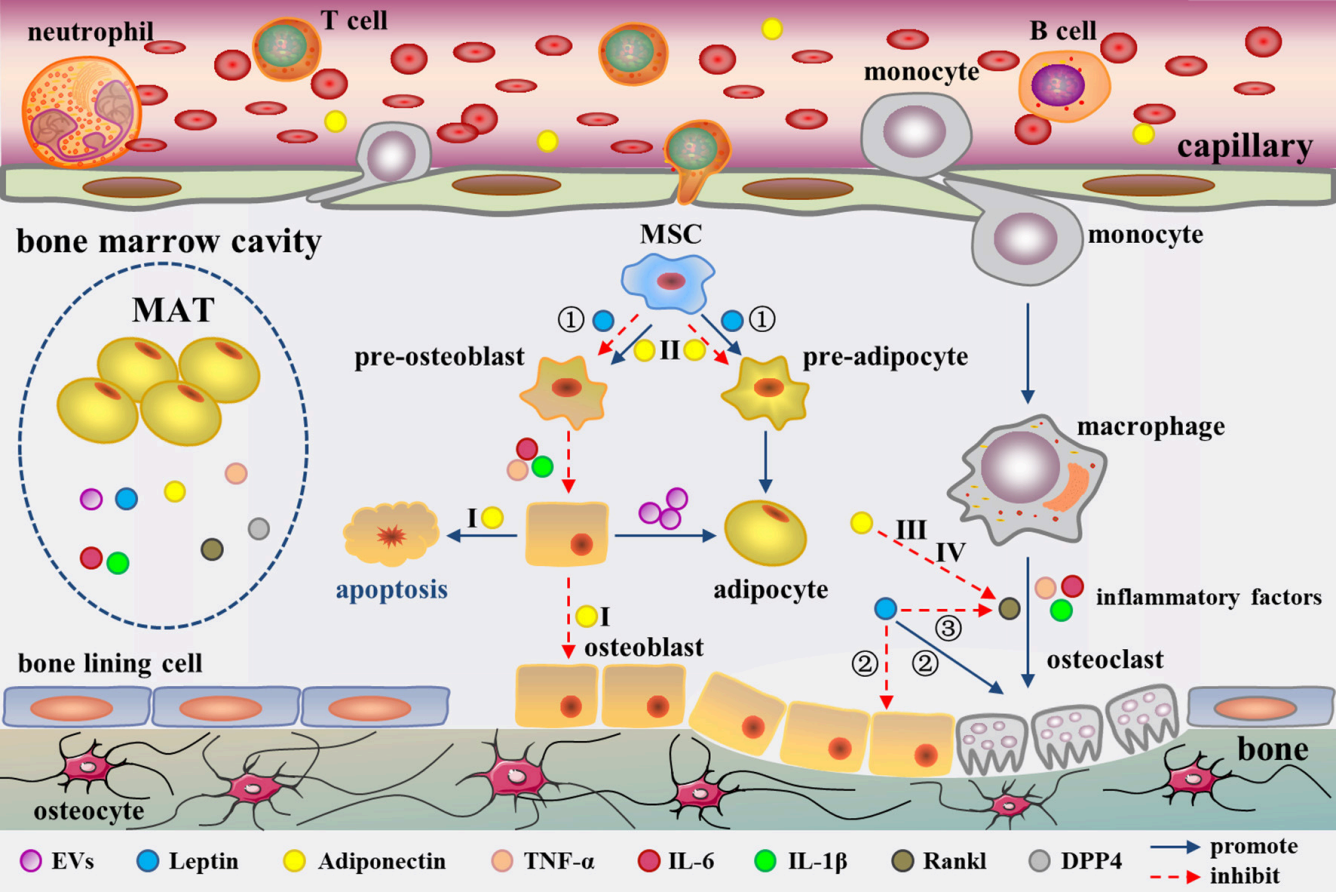
Secretion of MAT as well as adipocyte-derived molecules in the regulation of bone metabolism in the bone marrow cavity. MAT could secrete EVs, leptin, adiponectin, inflammatory factors, RANKL, and DPP4. These factors regulate bone metabolism from different aspects. Among them, adiponectin has been confirmed to enter into the circulation. EVs cause a phenotypic transformation of osteoblast to adipocyte. Leptin regulates bone metabolism in three ways (①-③). Moreover, Adiponectin regulates bone metabolism through four pathways (I-IV). Inflammatory cytokines promote osteoclast formation and adipocyte differentiation. RANKL promote osteoclast formation. The role of DPP4 in bone marrow cavity remains unclear.

The adipogenic/osteogenic differentiation of MSCs has always been considered to affect bone metabolism. In fact, MSC differentiation and even bone metabolism could be directly regulated by mature BM fat cells. Human MSC-derived osteoblasts demonstrated an elevated adipogenic profile and reduced osteogenic markers such as osteocalcin (OC) upon co-culturing with human MSC-derived adipocytes in the early study (79). In recent years, the same research group has explored the mechanism underlying this modulation. Adipocytes have been described as liberating extracellular vesicles (EVs) (80) (Figure 2). However, the definition of EVs is still lacking. It is conventionally believed that EVs are heterogeneous in size, encompassing the so-called microparticles/microvesicles (>100 nm) and exosomes (< 100 nm) in diameter (81, 82). The EVs from the human MSC-derived adipocytes were observed ~30–100 nm in size under transmission electron microscopy, but their protein profile remains to be characterized to classify (80). The EVs contain adipocyte specific transcripts e.g., Pparγ, leptin, Cebpα, Cebpδ, and anti-osteoblastic miRNAs including miR-138, miR-30c, miR-125a, miR-125b, and miR-31 (80). These EVs are probably involved in the down-regulation of osteogenesis in the co-culture system. Early studies have demonstrated that adipocytes have the ability to secrete exosomes (83, 84). Thus, the EVs in this study should be more accurately called exosomes. The evidence suggests that BM fat cells impact the phenotype of osteoblasts through paracrine of adipogenic transcripts and anti-osteoblastic miRNAs.

### Leptin

Leptin and adiponectin are known for their role in the regulation of global energy metabolism from 1990s (8, 85–88). Leptin is closely correlated with obesity, which forms a sense of satiety in the brain and reserves energy in peripheral tissues via leptin receptor (LepR) (89). Expression of leptin in primary culture system of human BM adpocytes was first confirmed in 1998 (88). Later studies proved that human MSC-derived mature adipocytes and human primary BM adipocytes can express and secrete leptin (90–94). Moreover, Liu et al. compared the expression of leptin in MAT and epididymal WAT (eWAT) in male C57BL/6J mice by microarray analysis. Their results showed that leptin is expressed at lower level in MAT than in eWAT (95). However, MAT expresses the similar level of leptin as iWAT and perirenal WAT (pWAT) in rabbits, while MAT secretes distinct concentration of leptin from WAT in humans (8). Nevertheless, the different secretion volume of leptin between MAT and WAT has not been studied in depth. It remains to be answered whether MAT could release more metabolic leptin that the traditional WAT.

LepR is highly expressed on MSCs (96). Previous research has suggested that leptin directly inhibits adipogenic differentiation and enhances osteogenesis in MSCs (97). Central or peripheral administration of leptin notably decreases the size and number of BM adipocytes in the leptin-deficient ob/ob mice (98–100). Besides, leptin treatment also blunts marrow fat in type 1 diabetic mice and calorie-restricted mice (101, 102). Compared to earlier studies, researchers have gotten different results recent years. Yue et al. found that limb bones exhibited a lower adipogenesis with conditional deletion of LepR from limb MSCs using Prx1-Cre, but not from the axial skeleton or hypothalamic neurons (103). Further studies suggested leptin increased adipogenesis by activating Janus kinase (Jak) 2/signal transducer and activator of transcription (Stat) 3 signaling pathway in MSCs (103) (Figure 2). The conflicting results may be resulted from the different animal models. A global gene knockout mouse model was used in the previous studies. However, the animal model with conditional deletion of leptin/LepR signaling in MSCs of limb bones was used in recent years, which is more intuitive and rigorous for exploring the role of leptin in MAT.

### Adiponectin

Since its discovery in 1995, adiponectin has gradually been considered as a biomarker for increased risk of insulin resistance (IR), cardiovascular diseases, bone loss, and certain cancers (104). Although adiponectin is derived from adipocytes, the plasma concentration of adiponectin is paradoxically decreased during obesity (105). As early as 2003, Delporte et al. have found that the plasma level of adiponectin is elevated in women with anorexia nervosa (106). Adiponectin is expressed in adipocytes derived from human and mouse MSCs (107, 108) and human MSC-derived adipocytes can secrete adiponectin (91, 109). However, the results from different groups showed diverse amount of adiponectin produced by BM adipocyte or MAT. Compared to peripheral adipocytes, mature human MSC-derived adipocytes, and human primary BM adipocytes express lower level adiponectin (110), which has also been confirmed in primary BM adipocytes from mice (95). In addition, the amount of adiponectin secreted by MSC-derived adipocytes is reported to be very low (94), even after stimulated with glucocorticoids (111, 112). However, Cawthorn et al. demonstrated that adiponectin secretion is greater from MAT than from WAT in conditions such as anorexia nervosa and cancer therapy in humans (8, 13). This discrepancy could be related to the fact that some groups analyzed the isolated BM adipocytes while Cawthorn et al. studied the intact MAT, which has been discussed before (7). It is undeniable that MAT has the ability to express and secrete adiponectin which can be released into the circulation (8, 30) to modulate systemic metabolism through endocrine effects (Figure 2). In addition, osteoblasts and osteoclasts are both in close contact with adipocytes in the BM, which creates a favorable topographic distribution for the crosstalk among them.

### Inflammatory Factors

The theme of MAT related pro-inflammatory factor has been of great significance. Compared with the epididymal white adipocytes, an independent transcriptomic study has revealed a unique phenotype for BM adipocytes characterized by higher levels of inflammatory response genes, such as tumor necrosis factor (TNF)-α, interleukin (IL)-6, and IL-1β (95) (Figure 2). The expression of TNF-α and IL1-β in BM adipocytes was also higher than that of visceral adipocytes under normal diet in C57BL/6J mice. Of note, mRNA level of pro-inflammatory cytokines was increased in the visceral adipocytes, while it was decreased in the BM adipocytes in high fat-induced obese mice (113). This suggests that MAT does not show the similar inflammatory response as WAT when stimulated by HFD. However, another research reported that human BM adipocytes in primary culture secrete only little amounts of IL1-β and TNF-α, but significant levels of IL-6 (114). Whatever, the BM adipocytes have a great potential to secrete pro-inflammatory factors, which may regulate bone metabolism (115, 116) and haematopoiesis (117) through paracrine, as well as affect the whole body metabolism by entering into the circulation.

### RANKL and DPP4

Fan and collaborators revealed BM adipocytes secrete receptor activator of nuclear factor kappa B ligand (RANKL) (77). In this study, they generated a mouse model with PTH1R deleted in the bone MSCs. They found that Pref-1 (pre-adipocyte marker) and RANKL tag synchronously were up-regulated in B220 (B cell lineage marker) negative cells in the knockout mice by flow cytometry. In addition, mRNA expression of RANKL is remarkable higher in the isolated marrow adipocytes than the whole BM. The above evidence suggests that MAT secretes RANKL (77) (Figure 2). Moreover, primary human BM adipocytes can also express RANKL to promote osteoclast differentiation by co-cultures of pre-osteoclasts with BM adipocytes (118).

As we know, dipeptidyl peptidase (DPP) 4 is a protease and its inhibitors have been widely used for the treatment of T2D (119). CD26, the membrane-bound form of DPP4, is enriched on the surface of adipogenic cell populations, but not osteochondrogenic progenitor cells. The shed CD26, also called DDP4, enters into the BM sera after adipogenic differentiation (36). The amounts of DPP4 are increased in distal tibiae with aging (36). These findings imply MAT may be involved in glucose metabolism through secreting DDP4. Overall, more potential of MAT for secreting factors remains to be clarified.

## MAT and Metabolism

### MAT and Bone Metabolism

As early as 2001, Justesen et al. found an age-related increase in MAT and decline in trabecular bone volume by iliac crest bone biopsies (120). Compared with age-matched controls, patients with osteoporosis exhibited an increased MAT (120), which suggests a strong correlation between osteoporosis and MAT. Furthermore, in diabetic mice, the trabecular bone loss is significantly correlated with the increased BM adiposity (121). In the young people, MAT was found to be negatively correlated with the amount of bone in the axial and appendicular skeleton (122, 123). Similar performance also occurs in the pelvis of the elderly (124) and children (125). In African-American and Caucasian men and women, a negative relationship existed between MRI-measured MAT and hip and lumbar BMD (126, 127). However, opposite result has revealed a positive relationship between MAT and bone mineral content (BMC) in white and non-Hispanic black girls aged at 4–10 years (128). Subjects with metabolic disorders such as morbid obesity and T2D have higher MAT but also higher femoral neck BMD (45). In short, most studies have shown a negative relationship between MAT and BMD in humans. But some studies suggest that BMC or BMD is positively associated with MAT, which reveals the relationship between marrow adiposity and bone mass is not a simple inverse correlation.

The communication between MAT and bone is complex. Factors including EVs, adipokines (leptin and adiponectin, etc.), inflammatory factors (IL-6 and TNF-α, etc.), and RANKL derived from MAT can regulate bone metabolism. EVs, containing adipocyte specific transcripts, down-regulate osteogenesis (79, 80). Leptin regulates bone metabolism at least in three ways. First, leptin decreases bone formation by activating Jak2/Stat3 signaling in MSCs (103). Second, leptin increases sympathetic activity through an indirect way depending on inhibition of 5-HT synthesis in raphe nuclei of brainstem (129, 130). The sympathetic signaling strengthens bone resorption through an ATF4-mediated process, and reduces bone formation through a CREB-mediated process (131, 132). Third, leptin also directly stimulates LepR in hypothalamic arcuate nuclei neurons and elevates Cart (cocaine amphetamine regulated transcript) expression, which decreases RANKL expression via an unknown mechanism, and thereby decreases bone resorption (133) (Figure 2).

The effect of adiponectin on bone metabolism presents a confusing situation. Serum adiponectin is negatively correlated with BMD in young healthy men (134), male hemodialysis patients (135), and men with spinal cord injury (136). In addition, adiponectin is also associated with decrease of bone mass in childhood (137). However, studies have shown that circulating level of adiponectin is not related with BMD in postmenopausal women (138, 139). Significant decrease of BMD was observed in adiponectin-knockout mice (140), suggesting a positive effect of adiponectin on bone geometry and density. Adiponectin regulates bone metabolism through four pathways. First, adiponectin signals could activate p38/mitogen-activated protein kinase (MAPK) pathway to increase RANKL expression in osteoblast through adiponectin receptor (AdipoR) 1 (141). Moreover, another signaling phosphoinositide 3-kinase (PI3K)/AKT pathway in osteoblasts is also induced simultaneously, resulting in inhibition of forkhead box protein O (FoxO) 1 and decrease of osteoblasts proliferation, as well as increase of osteoblasts apoptosis (142). The synergistic effect is to reduce osteogenesis and increase bone resorption, leading to decreased bone mass. Second, in MSCs, adiponectin serially activates AdipoR1, p38/MAPK, and the c-Jun signaling pathway to induce cyclooxygenase (COX) 2 expression (143). In this way, the adipocyte differentiation of MSCs is inhibited (144) and osteogenic differentiation is promoted. Third, in pre-osteoclast, adiponectin treatment significantly induces Appl1-mediated down-regulation of AKT1 activity and removes glycogen synthase kinase (GSK)-3β-mediated phosphorylation of nuclear factor of activated T cells (NFAT)2, giving rise to expressions of NFAT2-targeted genes decreased (145). The activation of this pathway leads to the inhibition of RANKL-induced osteoclastogenesis and increase of bone mass. Forth, adiponectin signals decrease the sympathetic tone also through FoxO1 in neurons of the locus coeruleus, further lower expression of RANKL, thereby inhibiting bone resorption (142). In the four signaling pathways above, the first one reduces bone mass while the other three increase bone mass. Previous study has suggested that adiponectin regulates bone mass via opposite central and peripheral mechanisms through FoxO1 (142) (Figure 2). It is unclear that the net effect of adiponectin on bone mass. Under different pathophysiological conditions, there must be a dominant pathway for adiponectin signal transduction.

The deletion of inflammatory factors like IL-6 and TNF-α has been reported to be a protective effect on HFD-induced trabecular bone loss (115, 116), but the molecular mechanisms is unclear. In addition, other adipocyte-secreted molecules, including chemerin (146–149), resistin (150, 151), visfatin (152), and omentin-1 (153, 154) have also been shown to modulate bone metabolism. But it is not clear if they are also expressed in BM adipocytes. Of course, only certain factors play a dominant role in regulating bone metabolism under specific conditions. This partly explains why adipocytes and osteoblasts share common precursor (39), but they are not always in a trade-off relationship. There are few studies on the molecular mechanism of MAT affecting bone metabolism. Future studies should pay more attention to it in order to predict the positive or negative regulation of MAT on bone metabolism under different pathophysiological conditions.

### MAT and Energy Metabolism

#### MAT as an Energy Depot in Bone

WAT reflects the high capacity of storing lipids. However, the transfer of lipids from WAT to other depots reflects the ability of retaining lipids is not a unique feature of WAT. Marrow adipocytes store significant quantities of fat and express insulin receptor (InsR) (34), they also respond to insulin-sensitizing anti-diabetic TZDs (54). This evidence tightly links MAT with the energy metabolism. Fatty acids and lipids can be used to generate adenosine triphosphate (ATP) for osteoblasts via the tricarboxylic acid cycle, although much less is known about their utilization (155). Studies have demonstrated that fatty acids can also be metabolized to generate ATP via Wnt activation in osteoblasts (155, 156). Although the relative concentrations and degree of saturation may be different (26, 157), fatty acids and lipids constantly circulate and are also present in BM sera, which favoring MAT as an energy depot in bone.

HFD in mice and obesity in humans promote expansion of bone MAT (45, 113), suggesting a stage of energy reserve. A recent research showed that HFD significantly increased expression of lipid storage marker fat-specific protein (FSP) 27, LepR and perilipin (PLIN) 5 in total bone tissue (33). This data suggests that there is an increase in lipid storage in MAT. In addition, running can improve bone quantity and quality while reduce the diameter and number of marrow adipocytes in obese mice (33). PLIN3 has been reported to play an important role in the β-oxidation of lipids as well as in promoting basal lipolysis (158, 159). Expression of PLIN3 is increased in MAT after running. In summary, these findings indicate that the marrow fat may be utilized as fuel to enhance bone formation.

#### MAT May Not Provide Energy During Acute Starvation

Nevertheless, other studies suggested that MAT is not a preferential position to provide energy. It seems that there is no connection between MAT and visceral adipose tissue (VAT) (160). During malnutrition, MAT does not decrease as a result of energy supply. Studies have shown that caloric restriction in animals and anorexia nervosa in humans lead to high marrow adiposity (53, 60). The incremental amount of MAT during nutritional deprivation may result from the differentiation of MSCs into adipocytes (8, 60). As early as 1979, Bathija et al. found that MAT is not catabolized during acute starvation (161). Moreover, the latest research has shown that MAT has the capacity to respond to β-adrenergic stimuli, however, its responses are muted in states of fasting and caloric restriction (162). This suggests that MAT may not participate in lipiolysis stimulated by β-adrenergic neuron to provide energy as peripheral WAT in the absence of energy.

### MAT and Glucose Metabolism

#### MAT and TZDs

TZD compounds, Pparγ agonists, have been widely used for the treatment of T2D and impaired glucose tolerance (IGT) with IR or hyperinsulinemia by reducing circulating free fatty acids (FFAs) and strengthening insulin sensitivity (163). TZDs stimulate Pparγ activation in adipose tissue and upregulate expression levels of genes involved in lipid metabolism, such as scavenger receptor CD36, fatty acid-binding protein (FABP) 4 and LPL (164, 165). Elevated expression of these genes promotes FFAs to translocate into adipose tissue, thus decreases serum FFAs concentration and eventually ameliorates IR. TZD-induced Pparγ activation contributes to MSCs differentiating into adipocytic lineage and is associated with bone loss and marrow adiposity, especially in aging female mice (56). These lines of evidence highlight the clinical observations that TZDs increase fracture risk in postmenopausal women (166, 167). Correspondingly, Pparγ inhibitor inhibits BM adiposity in mice after radiation exposure and in streptozotocin (STZ)-induced diabetic mice (168, 169). This evidence indicates the close relationship between MAT and glucose metabolism.

#### MAT Responds Differently to Insulin Stimuli

MAT may respond differently to insulin stimuli compared to peripheral WAT. Studies have shown that marrow adipocytes express InsR (34). In obesity and T2D, peripheral WAT develops IR and exhibits impaired insulin signaling (170). However, HFD-induced obesity did not impair insulin sensitivity in adipocytic progenitors of BM, as BM shows normal levels of pAKT as well as insulin signaling genes after insulin stimulation (113). This indicates that MAT has different insulin sensitivity from WAT.

#### MAT and IR

The augment of MAT has been observed in some conditions such as aging, and glucocorticoid-induced osteoporosis (GIOP), both of which are accompanied with IR (45, 171). Other results clearly showing a positive connection between MAT and glycated hemoglobin (HbA1c) or serum glucose concentrations (45, 160, 171). The adipocyte markers and marrow adiposity are increased in tibias in STZ-induced diabetic mouse model (121, 172). Moreover, patients with diabetes show a higher MAT compared to non-diabetic persons (173). In short, IR and diabetes status are strongly associated with increased MAT. However, no studies have investigated the molecular mechanisms as well as the crosstalk between MAT and glucose metabolism. We speculate MAT may regulate insulin sensitivity by secreting certain molecules like DPP4 and TNF-α. A latest research showed that hepatocyte DPP4 promotes VAT inflammation and IR in obesity (174). TNF-α is also involved in obesity-linked IR (175). Moreover, studies have shown that MAT can release DPP4 and TNF-α (36, 95). Thus, MAT may increase IR through secreting DPP4 and TNF-α, but more evidence is required to verify this view.

#### MAT and Insulin Sensitivity

Nonetheless, evidence also demonstrated there are no differences in MAT by diabetic status (176, 177). It has been summarized in the past that leptin and adiponectin can regulate food intake, lipid metabolism, glucose metabolism, etc. (178, 179). To our knowledge, MAT may secrete adiponectin and leptin to regulate blood glucose levels or insulin sensitivity. Previous study has shown that serum levels of adiponectin are positively associated with insulin sensitivity (105, 180). Insulin-resistant states including obesity and T2D are correlated closely to decreased adiponectin (105, 180, 181). Although adiponectin is increased in patients with type 1 diabetes (T1D), its levels are still positively correlated to insulin sensitivity (182). Studies have revealed that adiponectin can activate fatty acid oxidation and glucose uptake by increasing AMP-activated protein kinase (AMPK) phosphorylation and Pparα activity (183). A recent study of 50 obese and non-obese premenopausal women revealed a negative relationship between BM adiposity and IR, possibly mediated by increased secretion of adiponectin (184). In addition, it has been reported that more MAT content and adiponectin exist in the non-obese group (8, 184). Furthermore, leptin is increased in obesity inversely. The leptin signal is transmitted by Jak/Stat pathway to regulate food intake and body weight. In addition, PI3K signaling stimulated by leptin appears to take part in the decrease of blood glucose level (183). Therefore, adiponectin and leptin can increase insulin sensitivity and reduce blood glucose level, which is contrary to the effect of DPP4 and TNF-α derived from MAT. In brief, some studies have shown that MAT is positively related to IR and blood glucose levels, while some other studies have shown no correlation. More in-depth studies are needed to clarify the exact relationship.

## Concluding Perspectives

Substantial evidence have indicated that MAT secretes EVs, leptin, adiponectin, inflammatory cytokines such as IL-6 and TNF-α, RANKL, as well as DPP4. However, it has not been proved synchronously that these factors are both derived from BM fat cells and also regulate bone metabolism, energy metabolism and glucose metabolism. Since BM adipocytes and osteoblasts are derived from the same precursor cells, both of which exhibit a push-pull relationship in most conditions. Nevertheless, some studies have also shown a positive correlation between adipocytes and osteoblasts. The reason for these contradictory results has not been studied in-depth. The available evidence have manifested MAT can provide energy for osteoblasts during exercise, while the hypothesis of MAT acting as an energy supplier has been doubted as MAT is increased during caloric restriction and HFD. Animal and human studies have found that MAT and IR are inextricably linked. We infer that DPP4 and TNF-α secreted by MAT may increase IR, yet adiponectin and leptin increase insulin sensitivity. Because of the opposite effects of these hormones, different studies have yielded different results. As reviewed herein, MAT may affect global metabolism as a novel endocrine organ. Future studies will be critical to gain insight into the role of MAT and its relationship to bone and global metabolism.

## Author Contributions

XY designed this review. YL, YM, and XY wrote the manuscript.

### Conflict of Interest Statement

The authors declare that the research was conducted in the absence of any commercial or financial relationships that could be construed as a potential conflict of interest.
